# Network Pharmacology-Based Identification of Key Targets of Ziyin Mingmu Pills Acting on Age-Related Macular Degeneration

**DOI:** 10.1155/2023/5933125

**Published:** 2023-02-02

**Authors:** Yijing Yang, Ying Wang, Xiaoqing Liu, Ying Deng, Jing Lu, Feipeng Jiang, Fujiao Nie, Jun Peng, Qinghua Peng, Yuhui Qin

**Affiliations:** ^1^Hunan University of Chinese Medicine, Changsha 410208, China; ^2^Hunan Provincial Key Laboratory for the Prevention and Treatment of Ophthalmology and Otolaryngology Diseases with Traditional Chinese Medicine, Changsha 410208, China; ^3^Department of Ophthalmology, The First Affiliated Hospital of Hunan University of Chinese Medicine, Changsha 410007, China

## Abstract

**Objective:**

This study is designed to find out the molecular targets of effective Chinese medicine Ziyin Mingmu pills (ZMPs) in treating age-related macular degeneration (AMD) based on network pharmacology and experimental data.

**Methods:**

A comprehensive network pharmacology strategy that consists of three sequential modules (drug-disease target molecular docking, enrichment analysis, and external verification) was carried out to identify potential targets of ZMPs acting on AMD.

**Results:**

The active ingredients of ZMPs targeting 66 genes have effects on the process of AMD. GO and KEGG pathway enrichment analyses suggested that response to oxidative stress, regulation of angiogenesis, and lipid and atherosclerosis might serve as the most important signaling pathways in ZMPs for AMD treatment. Combined with the GSE29801 dataset for further analysis, two key genes, EGFR and VEGFA, were identified. Immune infiltration analysis showed that there was a strong association between EGFR and immune cell content. In addition, images were acquired following 24 h in the scratch experiment showed that ZMPs can reduce the percentage of wound healing distance. The Western blot assay found that ZMPs increased the expression of EGFR and decreased the expression of VEGFA.

**Conclusion:**

This study sheds light on some mechanisms of ZMP therapy for AMD, particularly the effect of ZMP on the oxidative stress in RPE and cell survival and angiogenesis in AMD. We propound ZMPs as a promising strategy to intervene in the process of AMD.

## 1. Introduction

Age-related macular degeneration (AMD) is one of the main causes of vision loss in the elderly [1]. AMD patients are mostly 50 years of age or older and are characterized by decreased central vision and blurred vision, which are progressively aggravated [2]. AMD can be divided into two types: dry AMD and wet AMD. The former is characterized by drusen and geographic atrophy with central dark spots and gradual vision loss [3], while the latter causes severe visual damage to patients through choroidal neovascularization, often causing macular hemorrhage, fibrous scarring, and irreversible blindness [4]. Many factors that are closely related to AMD have been discovered, such as age, oxidative stress, smoking, inflammation, and genetics [1, 5]. At present, there is no efficacy therapy for early AMD or dry AMD, and an antineovascular drug injection is the main method for clinical treatment of in advanced AMD or wet AMD, which have a positive impact on the restoration of vision and the improvement of prognosis [6, 7]. However, there are still unmet clinical needs for AMD therapy, and an intravitreal injection is a very big obstacle for patients with wet AMD [7]. On this basis, split-new treatment strategies need to be explored.

AMD belongs to the category of “faint vision (Shi Zhan Hun Miao in Chinese)” in traditional Chinese medicine (TCM) and attributes to depletion of essence, qi, and blood [8]. Modern medicine believes that ischemia and oxidative stress play an important role in the pathogenesis of AMD, which naturally followed the attribution of AMD in TCM [9, 10]. In clinical application, compared with the exposure to single-target drugs with side effects and poor therapeutic effects on complex diseases, TCM offers the advantages of multicomponents and multitargets with a synergistic strategy [11]. Ziyin Mingmu pill (ZMP), a Chinese patent medicine (Hunan Medicine Manufacturing Standard: Z20080745), is mainly composed of 16 kinds of medicinal materials: Gouqi (*Lycium barbarum* L.), Huangjing (*Polygonatum sibiricum* Delar. ex Redoute), Shudihuang (*Rehmannia glutinosa*), Tusizi (*Cuscuta chinensis* Lam.), Shanzhuyu (*Cornus officinalis* Sieb. et Zucc.), Mudanpi (*Paeonia suffruticosa* Andr.), Sanqi (Panax pseudo-ginseng Wall. var. notoginseng (Burkill) Hoo & Tseng), Danshen (*Salvia miltiorrhiza* Bunge), Niuxi (*Achyranthes bidentata* Blume), Danggui (Angelica sinensis (Oliv.) Diels), Chuanxiong (*Ligusticum chuanxiong* Hort.), Qianghuo (*Notopterygium incisum* Ting ex H. T. Chang), Shichangpu (*Acorus tatarinowii* Schott), Shanyao (*Dioscorea opposita* Thunb.), Fuling (*Poria cocos* (Schw.) Wolf), and Chushizi (*Broussonetia papyrifera* (L.) Vent.). Clinical observations have found the efficacy of ZMPs for AMD [12, 13]. However, the pharmacological mechanisms of ZMPs acting on AMD have not been completely elucidated.

## 2. Materials and Methods

### 2.1. Screening the Active Ingredients of ZMPs Acting on AMD

The traditional Chinese medicine system pharmacology (TCMSP, https://tcmspw.com/tcmsp.php) database is used to analyze the 16 kinds of medicinal materials of ZMPs. The TCMSP database collects 499 Chinese medicines from the Chinese Pharmacopoeia, containing 29,384 components, 3,311 targets, and 837 related diseases. The database uses predictive algorithms to obtain the relationship between drug targets and provides pharmacokinetic information including bioavailability (OB) and drug similarity (DL) for each compound; molecules with OB ≥ 30% or DL ≥ 0.18 are considered to be active ingredients [14]. In addition, the OMIM database, GeneCards database, and GEO database are used to screen the pathological targets of AMD. The Venn diagram is used to integrate the drug targets of ZMPs and the pathological targets of AMD to determine the mechanisms of action of potential targets.

### 2.2. GEO Data Acquisition

The GEO database (https://www.ncbi.nlm.nih.gov/gds/) stores microarray, next-generation sequencing, and other high-throughput sequencing data. We downloaded the GSE29801 Series Matrix File data file from the NCBI GEO public database. The annotation platform is GPL4133. There are 151 sets of transcriptome data, including the control group (*n* = 78) and the disease group (*n* = 73), for later verification.

### 2.3. Screening the Target of ZMP Treatment for AMD

STRING online database 11.0 (https://string-db.org) was used to analyze compounds of Gouqi, Huangjing, Shudihuang, Tusizi, Shanzhuyu, Mudanpi, Sanqi, Danshen, Niuxi, Danggui, Chuanxiong, Qianghuo, Shichangpu, Shanyao, Fuling, and Chushizi, and the confidence score >0.4 was used as the cutoff standard to obtain a target function-related protein network and protein-protein interaction (PPI) network.

### 2.4. Annotation of GO and KEGG Functions

To better understand the functional target genes and obtain better insights into the stress-responsive genes and pathways, R package “Cluster Profiler” was used to annotate the target genes. The Gene Ontology (GO) and Kyoto Encyclopedia of Genes and Genomes (KEGG) were used to assess related functional categories. The GO and KEGG enrichment pathways with a *p* value and *q* value both less than 0.05 are considered as the significant criterion.

### 2.5. Drug-Ingredients-Gene Symbols-Disease Network Construction

NetworkAnalyzer in Cytoscape (version 3.7.1) is used to analyze the topological parameters in the Drug-Ingredients-Gene Symbols-Disease (D-I-G-D) network construction, such as the median degree and the maximum degree. We further visualize the generated PPI network through Cytoscape software, analyze the role of key genes in the occurrence and development of diseases, and explore the correlation between diseases and gene expression.

### 2.6. Immune Cell Infiltration Analysis

CIBERSORT is an analytical tool to provide an estimation of the abundances of member cell types in a mixed cell population using gene expression data [15]. The RNA-seq data on patients with AMD were analyzed by CIBERSORT to reveal the relative proportions of 22 immune-infiltrating cells and perform the spearman correlation analysis of gene expression and immune cell content. *p* < 0.05 is considered to be statistically different.

### 2.7. Gene Set Enrichment Analysis

Gene set enrichment analysis (GSEA) uses a predefined gene set, ranks the genes according to the degree of differential expression in the two types of samples, standardize the *Z*-score, and then checks whether the preset gene set is enriched at the ranking table. This study used GSEA to compare the signal pathway differences sorted according to the level of expression and explored the molecular mechanism of the core genes of the two groups of patients. The number of replacements was set to 1000, and the replacement type was set to phenotypes. Statistical analysis is mentioned in section 2.8.5. Data Processing and Statistical Analysis.

### 2.8. In Vitro Validation of the Experiment

#### 2.8.1. Preparation of ZMPs

Ziyin Mingmu pills (30 g) were completely crushed and mixed, and the mixture was decocted with 100°C distilled water two times at a final ratio of 1 : 9 (weight/volume). The water extract was centrifuged at 10000 ×g for 15 min, and the extract was filtered and concentrated. The final concentration of the ZMP extract is 1.2 g/ml.

#### 2.8.2. Cell Viability Assay

The ARPE-19 cell line was purchased from Jennio Biotech Co., Ltd. (Guangzhou, China) and cultured in complete culture media (Sigma-Aldrich RPMI 1640). The ARPE-19 cell model of oxidative stress damage was constructed by incubating the cells with 50 *μ*L (concentration of 200 *μ*mol/L) hydrogen peroxide (H_2_O_2_). Cell viability was determined by the CCK8 assay (Signalway Antibody, CP002). Briefly, adult retinal pigment epithelial cell line-19 (ARPE-19) cells were seeded into a 96-well plate and treated with ZMPs at the indicated concentrations for 24 h. 10 *μ*L of CCK8 solution was added to each well, and the culture plate was incubated in an incubator at 5% CO_2_ and 37°C for 4 hours. After treatment, absorbance was measured at 560 nm using a microplate reader. Cell viability was calculated according to the absorbance of each well with the following formula: cell viability (%) = [(A560 sample − A560 blank)/(A560 control − A560 blank)] × 100%.

#### 2.8.3. Wound Healing Assay and Analysis (Scratch Experiment)

Cells were divided into three groups, including the normal group, model group, and ZPM group, and seeded into 6-well plates at 2 × 10^5^ cells/mL and incubated for 24 h. After the cells reached 100% confluence, the model group and ZPM groups were treated with H_2_O_2_ for 24 h. Wounds were generated using a 1 mL tip. Media were removed, and cells were washed with 600 *μ*L of PBS. Then, 600 *μ*L of the complete culture media was added to the normal group and the model group, and 400 *μ*L of the complete culture media and 200 *μ*L of the ZMP extract were added to the ZMP group. Images were acquired following media replacement and 24 h.

#### 2.8.4. Western Blot Assay

ARPE-19 cells were divided into the normal group, model group (treated with 50 *μ*L H_2_O_2_, dynamics of H_2_O_2_ availability to ARPE-19 cultures in models of oxidative stress), and ZMP group (treated with 200 *μ*L of ZMP extracts). After treatment, cells were collected and lysed for 30 min on ice with lysis buffer. Samples were centrifuged at 15,000 rpm for 10 min at 4°C, and total protein concentrations were measured and then denatured. Aliquots of 20–40 *μ*g of lysates were separated in a 6–12% sodium dodecyl sulfate polyacrylamide gel along and transferred onto a polyvinylidene difluoride membrane preactivated by methanol. The membrane was blocked for 2 h at room temperature with 5% nonfat milk powder dissolved in TBST (0.1% Tween-20 in TBS) and then incubated with primary antibodies against EGFR (Abcam, ab52894), VEGFA (Proteintech, 66828-1-Ig), or beta-actin (Proteintech, 66009-1-Ig) for 12 h at 4°C. Secondary antibodies were diluted in TBST containing 5% milk and incubated for 1 h at 25°C. The immune-reactive targets were detected by using the ECL western blotting substrate kit. Band density was analyzed by using ImageJ software and normalized with the internal control.

#### 2.8.5. Data Processing and Statistical Analysis

SPSS 26.0 software combined with GraphPad Prism 9 software was used for data processing and analysis. The enumeration data were analyzed by the chi-square test. For the comparison of quantitative data between two groups, the normality test was first performed. If the normality of each group was satisfied and the variance between the two groups was equal, the *t*-test was used for the comparison between groups; otherwise, the nonparametric Wilcoxon rank-sum test was considered. For the comparison between multiple groups, if the continuous data obeyed the normal distribution and the variance was homogeneous, the one-way analysis of variance (ANOVA) was used for the comparison between the groups; LSD was used for the post hoc test. If the difference between the groups was statistically significant, the Bonferroni method was used for the pairwise comparison. Subject to normal distribution or unequal variance, the Kruskal–Wallis rank-sum test was used for comparison between multiple groups. When there was a statistical difference between groups, the DSCF method was used for multiple comparisons. *p* < 0.05 was considered a statistically significant difference.

## 3. Results

### 3.1. Screening the Target of ZMPs Acting on AMD

**Figure 1:**
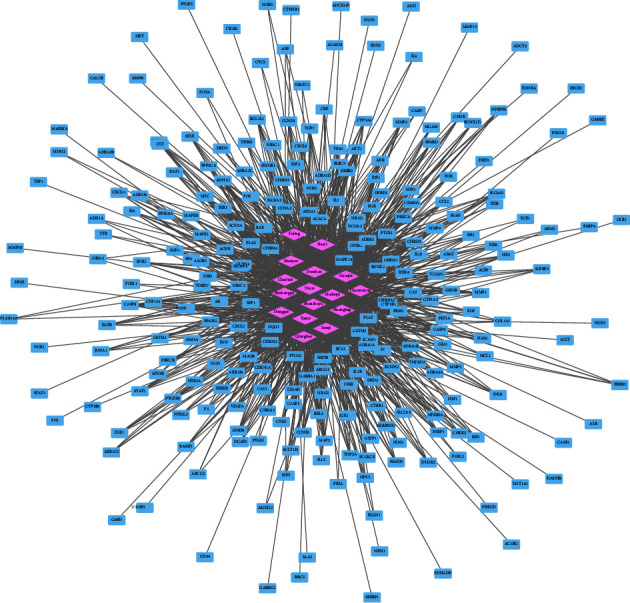
There are a total of 246 targets that 16 different Chinese medicine active ingredients of drugs act on. Red labels represent 16 different Chinese medicines. Blue labels represent 246 targets of actions of 16 different Chinese medicine active components.

**Figure 2:**
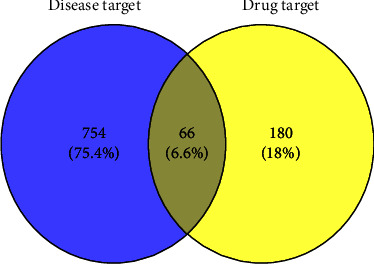
The 66 overlapping genes between disease targets and drug targets. The blue circle indicates the targets of AMD disease, and the yellow circle indicates the drug targets of ZMP active ingredients.

The method of network pharmacology is used to analyze and determine the effect of Gouqi, Huangjing, Shudihuang, Tusizi, Shanzhuyu, Mudanpi, Sanqi, Danshen, Niuxi, Danggui, Chuanxiong, Qianghuo, Shichangpu, Shanyao, Fuling, and Chushizi, a total of sixteen compound Chinese herbal medicines, on AMD. We explored 820 targets related to AMD by the OMIM database and the GeneCards database (relevance score≥5). With bioavailability (OB) ≥ 30% and drug-like properties (DL) ≥ 0.18 as the threshold, we screened these sixteen Chinese medicines from the TCMSP database, and 246 targets that were the active ingredients of the medicines acting on AMD were obtained. We used Cytoscape to show the relationship between Chinese medicine components and targets in the form of a network diagram (Figure 1). The 246 drug targets and 820 disease targets related to AMD were intersected, and 66 intersecting targets were disclosed (Figure 2).

### 3.2. Annotation of Target Gene Function Enrichment

**Figure 3:**
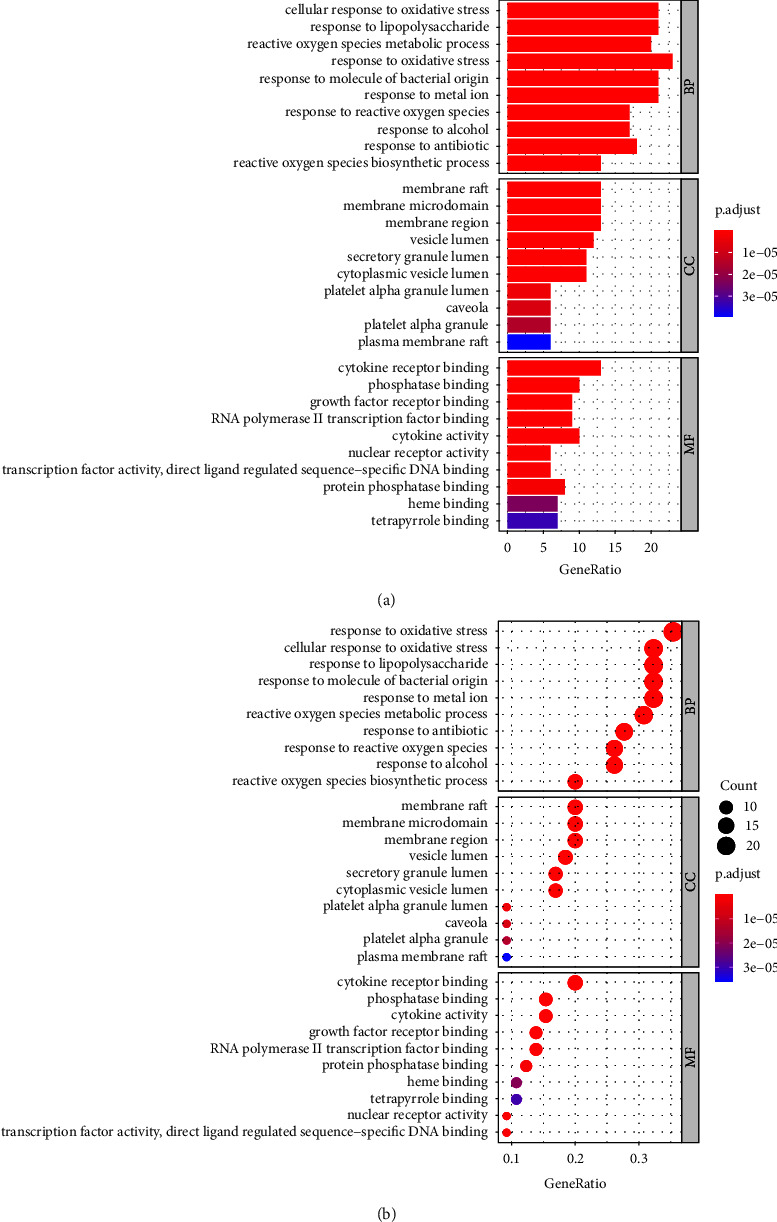
GO analysis shows the top 10 terms of the 66 overlapping genes involved in AMD. The *Y*-axis in (a) and (b) represents the pathways. The *X*-axis in (a) indicates the number of genes enriched for the term, while the *X*-axis in (b) indicates the gene ratio of 66 overlapping genes. The redder the color, the smaller the value of *p*.adjust. This also means greater credibility and more importance. In contrast, the bluer the color, the greater the value of *p*.adjust, *p*.adjust < 0.05.

**Figure 4:**
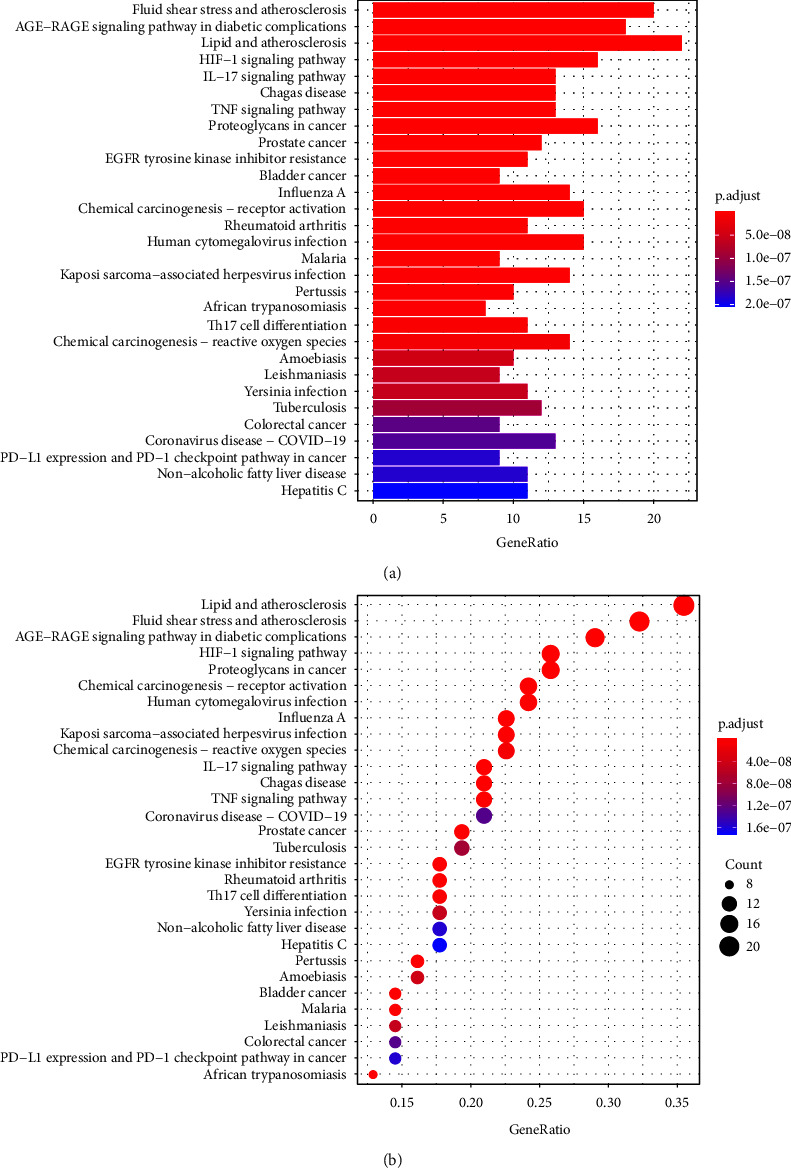
The KEGG pathway enrichment analysis shows the top 30 terms. The *Y*-axis in (a) and (b) represents the pathways. The *X*-axis in (a) indicates the gene count enriched for the term, while the *X*-axis in (b) indicates the gene ratio of 66 overlapping genes. The redder the color, the smaller the value of *p*.adjust. This also means greater credibility and more importance. In contrast, the bluer the color, the greater the value of *p*.adjust, *p*.adjust < 0.05.

By exploring the network pharmacology and pathway relationship between disease targets and drug targets, we further inquire into the potential mechanism of Chinese herbal compounds influencing age-related macular degeneration. Using R package “Cluster Profiler,” the 66 intersecting targets were used for GO enrichment and KEGG pathway analyses. The GO enrichment results showed that the main pathways have cellular response to oxidative stress, response to lipopolysaccharide, reactive oxygen species metabolic processes, and response to oxidative stress (Figure 3). The KEGG enrichment results showed that the main pathways involved in 66 genes are fluid shear stress and atherosclerosis, AGE-RAGE signaling pathways in diabetic complications, lipid and atherosclerosis, HIF-1 signaling pathways, and other signaling pathways (Figure 4).

### 3.3. D-I-G-D Network Construction

**Figure 5:**
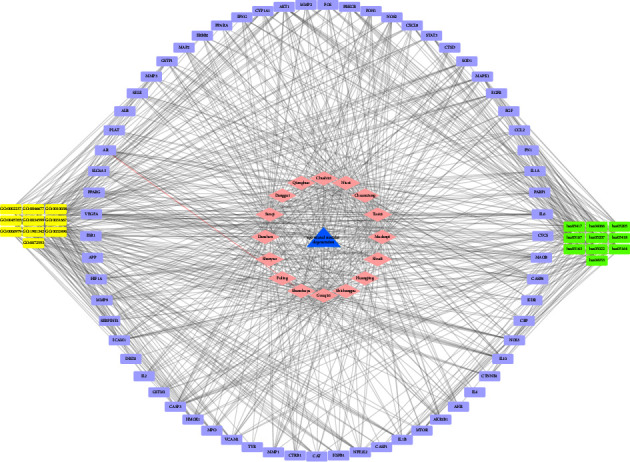
Drug-ingredients-gene symbols-disease (D–I–G–D) network construction. The networks showed the molecular mechanisms of 16 compound Chinese medicines regulating 66 targets and their effects on AMD.

We further used Cytoscape software to generate a network visualization of Chinese medicines against age-related macular degeneration targets and an interactive diagram of core target-related pathways. The chart can clearly show the molecular mechanisms of 16 compound Chinese medicines regulating 66 targets and their effects on AMD (Figure 5).

### 3.4. External Verification

**Figure 6:**
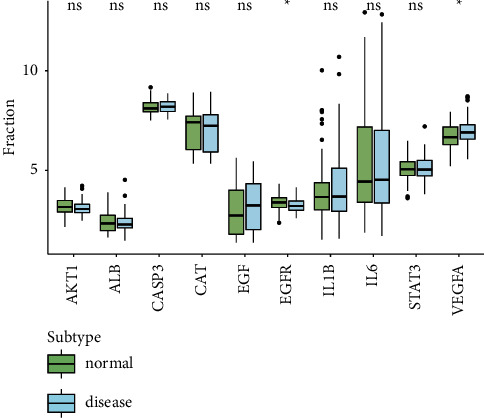
The external verification of the top 10 genes in patients with AMD. Data were from GEO dataset GSE29801 dataset, and EGFR and VEGFA were identified as key genes in the process of AMD. ^*∗*^*p* value <0.05.

We obtained the 66 intersecting target proteins through the string database and then selected the top ten genes of degree through Cytoscape. Then, we used the GSE29801 dataset for external verification and identified two key genes, epidermal growth factor receptor (EGFR) and vascular endothelial growth factor A (VEGFA), for follow-up research (Figure 6).

### 3.5. Immune Infiltration Analysis in AMD

**Figure 7:**
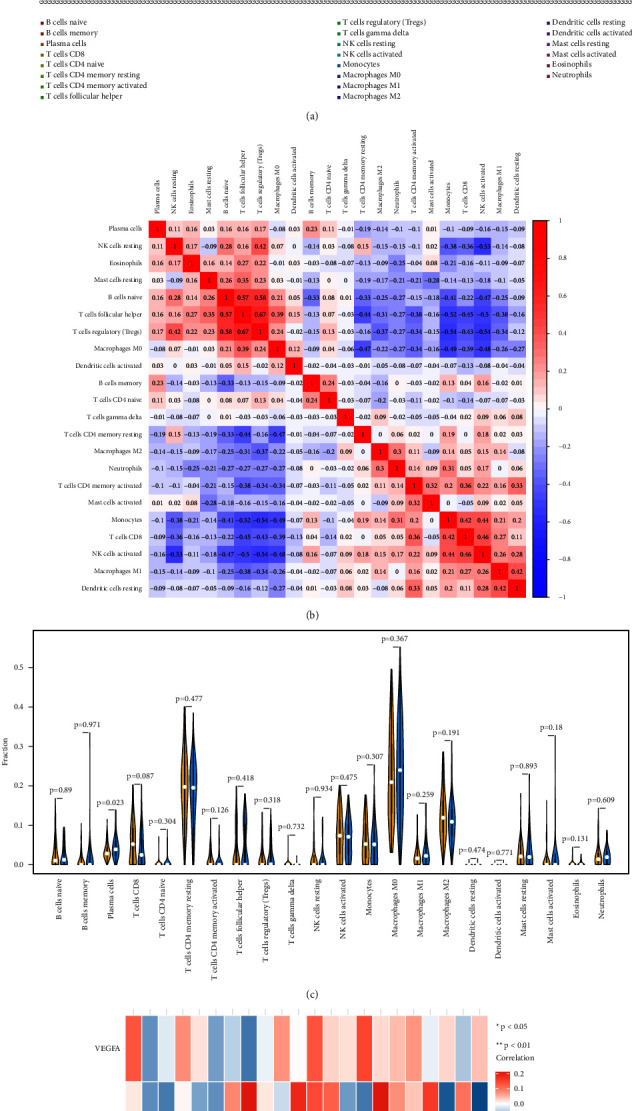
Estimation of fractions of immune cells. (a) 22 immune cells were annotated by various colors. (b) The heatmap illustrating the differences in infiltrating immune cells, and the colors ranging from blue to red represent the infiltration density from low to high. (c) The Wilcoxon rank-sum test was used to accurately compare the differences, and the results showed that plasma cells (*p* = 0.023) in the high-risk group (blue symbol) displayed a significantly higher infiltration density. (d) The relationship between 2 hub genes and 22 infiltrating immune cells was evaluated, and there was a strong association between EGFR and immune cell content. ^*∗*^*p* value <0.05; ^*∗∗*^*p* value <0.01.

We hereby analyze the relationship between key genes and immune infiltration in the AMD dataset and investigate the potential molecular mechanisms of key genes affecting the AMD's immune microenvironment. Compared to the normal group, the results represented that plasma cells in patients with AMD were significantly higher, see Figure 7.

### 3.6. Gene Set Enrichment Analysis Enrichment

**Figure 8:**
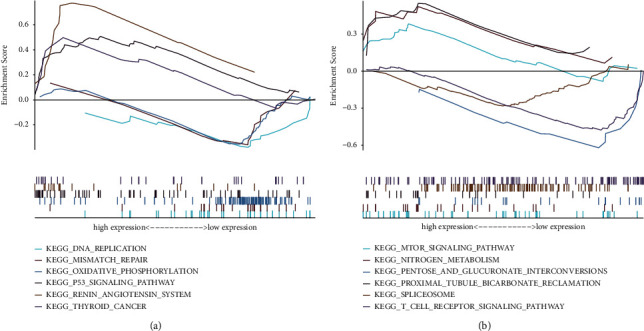
Pathways enrichment involved in EGFR and VEGFA. Multienrichment GSEA showed that EGFR was mainly enriched in signal pathways such as KEGG_OXIDATIVE_ PHOSPHORYLATION, KEGG_P53_SIGNALING_PATHWAY, and KEGG_DNA_ REPLICATION. VEGFA was mainly enriched in pathways including KEGG_MTOR_ SIGNALING_ PATHWAY, KEGG_T_CELL_RECEPTOR_ SIGNALING_ PATHWAY, and KEGG_ SPLICEOSOME.

We studied the specific signaling pathways involved in two key genes, EGFR and VEGFA, and explored the potential molecular mechanisms of core genes affecting the progression of AMD. The results of multienrichment GSEA showed that EGFR was mainly enriched in signal pathways such as KEGG_OXIDATIVE_ PHOSPHORYLATION, KEGG_P53_SIGNALING_PATHWAY, and KEGG_DNA_ REPLICATION. VEGFA was mainly enriched in pathways including KEGG_MTOR_ SIGNALING_PATHWAY, KEGG_T_CELL_RECEPTOR_ SIGNALING_PATHWAY, and KEGG_ SPLICEOSOME (Figure 8).

### 3.7. Experimental Validation In Vitro

**Figure 9:**
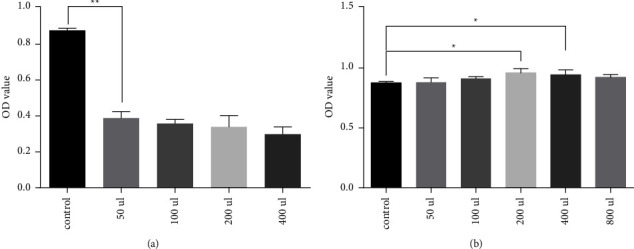
Detection of ARPE-19 cell viability after treatment with different doses of hydrogen peroxide (a) and ZMP extract (b) using one-way ANOVA statistical analysis. ^*∗*^*p* < 0.05; ^*∗∗*^*p* < 0.01.

The effect of ZMPs on AMD was validated in vitro. We first examined the effect of ZMPs on the viability of ARPE-19 cells, which are the commonly used AMD-cell models. The results of CCK8 detection of different doses of hydrogen peroxide-induced cell damage and ARPE-19 cell activity after treatment with ZMP extracts showed that the dose of 50 *μ*L hydrogen peroxide in the 96-well plate was the best choice and that the ZMP extract dose was 200 *μ*L. The statistical results are shown in Figure 9.

**Figure 10:**
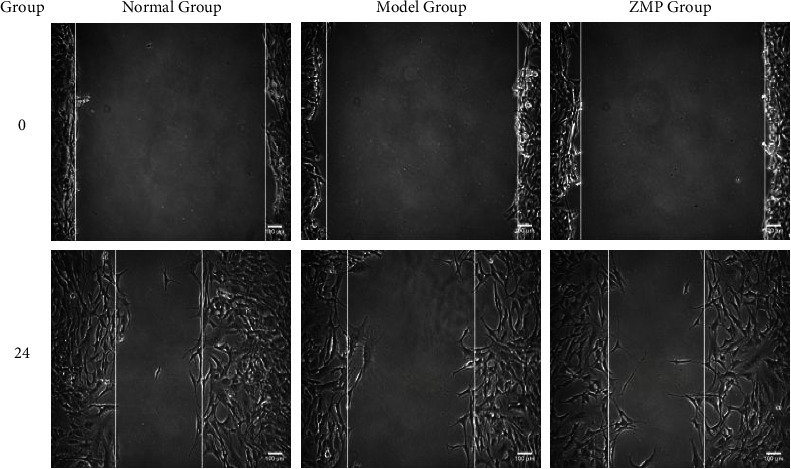
The morphology of ARPE-19 cell migration in each group. Normal group, nontreated cells; model group, cells treated with 50 *μ*L of H_2_O_2_ for 24 h before scratch; ZMP group, cells scratched and then treated with 200 *μ*L of the ZMP extract for 24 h.

**Figure 11:**
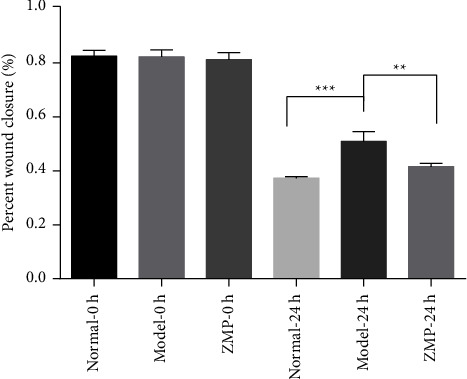
Comparison of ARPE-19 cell migration in each group. H_2_O_2_ significantly reduced cell migration, and the ZMP extract enhanced the migration. The mean (column) and standard deviation (error bars) of wound closure over time for nontreated and treated cells. One-way ANOVA statistical analysis; ^*∗∗*^*p* < 0.01; ^*∗∗∗*^*p* < 0.0001.

Compared to the model group by the scratch experiment, images were acquired following media replacement (*T* = 0 h), which displayed no difference in the percentage of wound healing distance in each group. Compared to the model group by the scratch experiment, images were acquired following 24 h, which showed that the percentage of wound healing distance in the ZMP group was significantly lower than that in the model group. There was no difference between the normal group and ZMP group, as shown in Figures 10 and 11.

**Figure 12:**
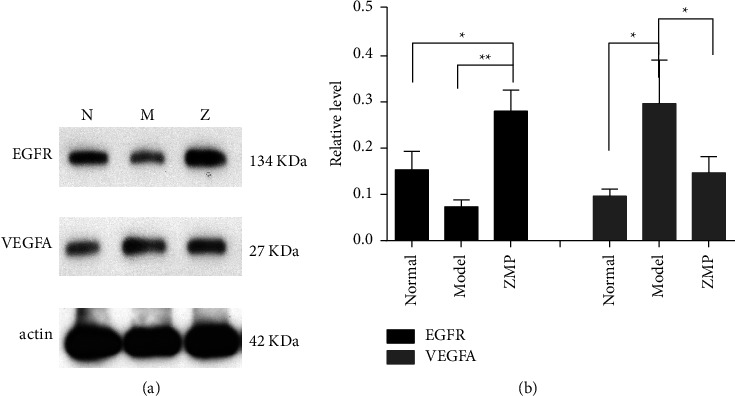
Comparison of EGFR and VEGFA protein expression in ARPE-19 cells in each group. (a) N represents the normal group, M represents the model group, and Z represents the ZMP extract group. (b) Compared to those of the ZMP extract group, the expression levels of EGFR in the normal group and the model group were decreased; compared to those of the model group, the expression levels of VEGFA in the normal group and the ZMP extract group were decreased. One-way ANOVA statistical analysis; ^*∗∗*^*p* < 0.01; ^*∗*^*p* < 0.05.

The expression levels of EGFR and VEGFA proteins in ARPE-19 cells in each group were detected by Western blot (see Figure 12). The exposed film was scanned and analyzed with the Quantity One grayscale analysis software. Compared to those of the ZMP extract group, the expression levels of EGFR in the normal group and the model group were decreased; compared to those of the model group, the expression levels of VEGFA in the normal group and the ZMP extract group were decreased. The statistical analysis results of the data are as follows.

## 4. Discussion

In aging or disease, the excessive accumulation of reactive oxygen species (ROS) induced by photoreceptors and RPE dysfunctions are considered the major causes of AMD [16, 17]. Chinese medicine is a style of traditional medicine, aims to regulate biological activity for balance, and has been used to treat many diseases for thousand years in China [18, 19]. Liver-kidney yin deficiency is considered to be the major disharmony in patients either with AMD, and ZMP has the effect of supplement deficiency and invigorate blood, which effectively improve the disharmony of liver-kidney yin deficiency [12, 20]. However, the specific pharmacological mechanism is incompletely understood. The oxidative injured ARPE-19 cell line was ideally characterized in vitro models of AMD [21]. In this study, we applied network pharmacology to investigate the mechanism of ZMPs acting on AMD, and the results showed active ingredients of ZMPs targeting 66 gene effects on the process of AMD. GO and KEGG pathway enrichment analyses suggested that response to oxidative stress, regulation of angiogenesis, and lipid and atherosclerosis might serve as the most important signaling pathways in ZMPs for AMD treatment. Immune infiltration analysis showed that there was a strong association between AMD and immune cell content. EGFR and VEGFA were identified as key genes of AMD, and ZMP increased the expression of EGFR, while it decreased the expression of VEGFA and reduced the percentage of wound healing distance in ARPE-19 cells.

Studies found that downregulated expression of vascular endothelial growth factor (VEGF) and EGFR can inhibit NV [22, 23]. In wet AMD, oxidative stress can alter RPE cells to express VEGF, in turn, induced choroidal neovascularization [24]. A study has shown that an increase in VEGFA is sufficient to cause both forms of AMD pathologies [25]. Brolucizumab that binds to the major isoforms of VEGFA has been administered as an effective treatment for neovascular AMD [26]. There are distinct anti-VEGF molecules available for AMD treatment based on their structure and function; however, the risk of intravitreal injections and economic cost burden still exists [27]. So far, treatment outcomes for dry AMD are still poor, with almost all patients exhibiting RPE dysfunction and loss [28]. EGFR is critical in cell proliferation, differentiation, and migration, which raise cell survival under oxidative stress. Studies on the ARPE-19 cell line indicate that oxidative stress induced by H_2_O_2_ suspends expression of EGFR, resulting in ARPE-19 cell loss. This finding suggests that EGFR may be an important target for preventing oxidative injury in RPE cells [29]. Another study in RPE cells has found that suppressing aberrant EGFR activation induced by cigarette smoking has an important effect on VEGF release [30].

It is known that multiple factors including the intraocular microenvironment affected the progression of AMD.The immune regulation of RPE cells in the intraocular microenvironment depends on the ability of RPE cells to express cell surface molecules and soluble inhibitors, such as transforming growth factor *β* (TGF- *β*), pigment epithelium-derived factor (PEDF), complement regulatory proteins (CD46, CD55, and CD59a), and interleukin (IL)-1 receptor antagonist [31, 32]. The immune microenvironment is mainly composed of immune cells, extracellular matrix, a variety of growth factors, inflammatory factors, and special physical and chemical characteristics, which significantly affect the diagnosis of diseases and the sensitivity of clinical treatment [33, 34]. Immune infiltration analysis suggested there was a strong association between EGFR and immune cell content, and the results were in line with expectations. In AMD, inflammation develops within the retina in an attempt to maintain ocular homeostasis and physiological allostasis, reflected by increased expression of anti-inflammatory cytokine IL-10 [35]. Furthermore, dysregulation of macrophage and activation of retinal microglia are observed and potentially contribute to macular degeneration [36, 37]. Our in vitro validation experiment showed that the ZMP extract promoted the proliferation and migration ability of ARPE-19 cell lines and was involved in the inhibition of induced angiogenesis.

There are some shortcomings in this study. The targets obtained by the network pharmacology of traditional Chinese medicine compound prescriptions are mainly obtained by the superposition of the predicted targets of each single traditional Chinese medicine composition. In the actual experiment and clinical medication process, different traditional Chinese medicine preparation methods and time will affect the efficacy of the drug, so experimental or clinical use. The composition of TCM supplements used clinically is more complex than that of single TCM ingredients. This study only qualitatively predicted and preliminarily verified the drug components of ZMPs and the targets of AMD disease, and the definite pharmacological effects still need to be verified through animal experiments and even clinical trials.

## 5. Conclusion

AMD is a complex multifactorial disease involving complex molecular signaling pathway networks during its course. This study sheds light on some mechanisms of ZMP extract therapy for AMD, particularly the effect of ZMP extracts on the oxidative stress in RPE and cell survival and angiogenesis in AMD. On this account, we propound the ZMP extract as a promising strategy to intervene in the process of AMD.

## Data Availability

The network pharmacology datasets used in the present study are all publicly available. Data gathered in this study are available from the authors upon request.
